# Identification of Potential Prognostic Biomarkers Associated With Macrophage M2 Infiltration in Gastric Cancer

**DOI:** 10.3389/fgene.2021.827444

**Published:** 2022-01-17

**Authors:** Baohong Liu, Xueting Ma, Wei Ha

**Affiliations:** ^1^ State Key Laboratory of Veterinary Etiological Biology, Key Laboratory of Veterinary Parasitology of Gansu Province, Lanzhou Veterinary Research Institute, Chinese Academy of Agricultural Sciences, Lanzhou, China; ^2^ Department of Radiology, Affiliated Hospital of Gansu University of Traditional Chinese Medicine, Lanzhou, China

**Keywords:** gastric cancer, prognosis biomarkers, macrophage M2, weighted gene co-expression network analysis, Protein-protein interaction network

## Abstract

Gastric cancer is a common cancer afflicting people worldwide. Although incremental progress has been achieved in gastric cancer research, the molecular mechanisms underlying remain unclear. In this study, we conducted bioinformatics methods to identify prognostic marker genes associated with gastric cancer progression. Three hundred and twenty-seven overlapping DEGs were identified from three GEO microarray datasets. Functional enrichment analysis revealed that these DEGs are involved in extracellular matrix organization, tissue development, extracellular matrix–receptor interaction, ECM-receptor interaction, PI3K-Akt signaling pathway, focal adhesion, and protein digestion and absorption. A protein–protein interaction network (PPI) was constructed for the DEGs in which 25 hub genes were obtained. Furthermore, the turquoise module was identified to be significantly positively coexpressed with macrophage M2 infiltration by weighted gene coexpression network analysis (WGCNA). Hub genes of *COL1A1, COL4A1, COL12A1*, and *PDGFRB* were overlapped in both PPI hub gene list and the turquoise module with significant association with the prognosis in gastric cancer. Moreover, functional analysis demonstrated that these hub genes play pivotal roles in cancer cell proliferation and invasion. The investigation of the gene markers can help deepen our understanding of the molecular mechanisms of gastric cancer. In addition, these genes may serve as potential prognostic biomarkers for gastric cancer diagnosis.

## Introduction

Gastric cancer (GC) is a malignant tumor originating from the epithelium of gastric mucosa and has the highest incidence rate among all types of malignant tumors in China (Kang et al., 2015). Although GC is a complex disease involving in multiple genes and pathways (Shiozaki et al., 2001; Carneiro et al., 2012; Ma et al., 2013), the exact molecular mechanisms of its development and prognosis need more investigations. Discovering new prognosis biomarkers and therapeutic targets of GC will aid in deeply understanding the development of GC and, thus, improving the life quality of patients. Given the development of high-throughput technologys, such as microarray and next generation sequencing, which can detect a whole genome simultaneously, numerous mRNA expression datasets have been produced for various biological purposes, facilitating the analysis of multiple genes (He et al., 2016; Li et al., 2019a). Microarray analysis for cancers has been widely used to identify cancer-related genes and pathways, allowing the mechanisms of cancer progression to be revealed to some extent (Sun et al., 2017). However, results from different experiments are not always consistent because of the heterogeneity of biological samples and the different detection platforms and data processing methods used (Gao et al., 2018). In the current study, we integrated differentially expressed genes (DEGs) from three different datasets to reduce the false discovery rate as much as possible. A series of bioinformatics analyses was performed on overlapping DEGs to explore a reliable basis for the molecular mechanisms of GC pathogenesis and identify the molecular markers for GC diagnosis.

## Material and Methods

### Data Availability and Preprocess

Datasets including both GC samples and controls were downloaded from the GEO database^1^ with accession numbers GSE54129, GSE79973, and GSE118916 (Table 1). Each dataset was preprocessed by: 1) removing probesets with no Entrez GeneID; 2) for one gene with multiple GeneID, preserving the probeset with the most sample frequency that having the maximum of expression values across probes; and 3) averaging the intensities if more than one probeset remained after the above steps (Liu and Cai, 2017).

**TABLE 1:** The GEO gene expression datasets description.

**GEO**	**Platform**	**Normal**	**Tumor**	**DEGs**
GSE54129	GPL570	21	111	2475
GSE79973	GPL570	10	10	767
GSE118916	GPL570	15	15	1838

### DEG Identification

DEGs were screened out by limma which is an R package (Smyth, 2004). We set the differential expression (DE) cutoff value to |log2 (FC)| ≥ 1 and adj. *p* < 0.05 for the three microarray datasets.

### Functional Enrichment Analysis

Gene functional enrichment for DEGs was implemented using the R package clusterProfiler (Yu et al., 2012). GeneMANIA^2^ was performed to create the interaction network for hub genes and other neighboring genes that interacted by physical interaction, gene coexpression, gene colocation, gene enrichment, or website prediction (Warde-Farley et al., 2010). ClusterProfiler (Yu et al., 2012) was conducted to perform gene set enrichment analysis (GSEA) with TCGA-STAD RNA-seq data including 232 STAD samples. These samples were further classified into two categories (High hub gene expression category vs Low hub gene expression category) by the median expression value of each hub gene. Differential expression analysis was then performed to the two categories of genes to get the DE measurements which are the input of the GSEA.

### Immune Cell Infiltration Prediction Using CIBERSORT

The cellular components of tissues were predicted by the CIBERSORT deconvolution algorithm based on the standardized gene expression profiles (Newman et al., 2015). The relative components of 22 infiltrating immune cells in each sample were examined by CIBERSORT.R^3^ using the three GEO expression datasets and the Leukocyte signature matrix (LM22) containing 547 genes’ expression matrix. *p* < 0.05 was set as the criteria for each sample, indicating that the predicted proportion of each infiltrating immune cell subtype is fairly accurate and suitable for further analysis.

### Protein-Protein Interaction Network Construction and Analysis

The database of STRING^4^ was explored to construct the protein–protein interaction (PPI) network for DEGs (Szklarczyk et al., 2011). And the plug-in of Cytoscape named Cytohubba (Chin et al., 2014) was used to identify the hub genes from the DEGs associated PPI network.

### Weighted Gene Coexpression Network Construction

WGCNA was performed by R package to construct the weighted gene coexpression network and to identify the coexpression modules (Langfelder and Horvath, 2008). The hclust function was applied to cluster the samples by hierarchical clustering algorithm. The soft thresholding power β was selected by the function of pickSoftThreshold when the scale free topology fitting indices *R* ^2.^ reached 0.9 to satisfy the scale-free characteristic for the biological network. Then the scores of topology overlap (TO) were calculated to create the network. dissTOM that is 1-TO was used as the measure of distance to cluster genes hierarchically in a dendrogram. Finally, a dynamic tree-cutting algorithm was applied to determine the assignments of modules. Module eigengenes (MEs) were calculated by the function of moduleEigengenes. And Pearson correlation coefficients between MEs and the macrophage M2 compositions were evaluated.

### Correlation Between Hub Genes and Tumor-Infiltrating Immune Cells Markers

Relationships between the hub genes’ expression level and the components of immune cell infiltration in GC were evaluated by TIMER (Tumor Immune Estimation Resource) database^5^ (Li et al., 2017). There are 10,897 samples with 32 cancers coming from TCGA database. It also includes a series of immune cells, such as CD4^+^ T cells, CD8^+^ T cells, B cells, neutrophils, macrophages, and dendritic cells.

### Survival Analysis

Survival analysis was performed to elucidate the relationship between the hub genes’ expression level and the prognosis of GC by Gene Expression Profiling Interactive Analysis^6^ (GEPIA) (Tang et al., 2017). It is a database that can evaluate survival outcomes for genes by using The Cancer Genome Atlas (TCGA) datasets. In addition, we tested the survival analysis results by microarray datasets using the Kaplan–Meier (KM) plotter^7^, which can evaluate the prognosis efficacy of genes on survival for multiple cancers (Szász et al., 2016). *p* < 0.05 was set as the significance criteria.

## Results

**FIGURE 1:**
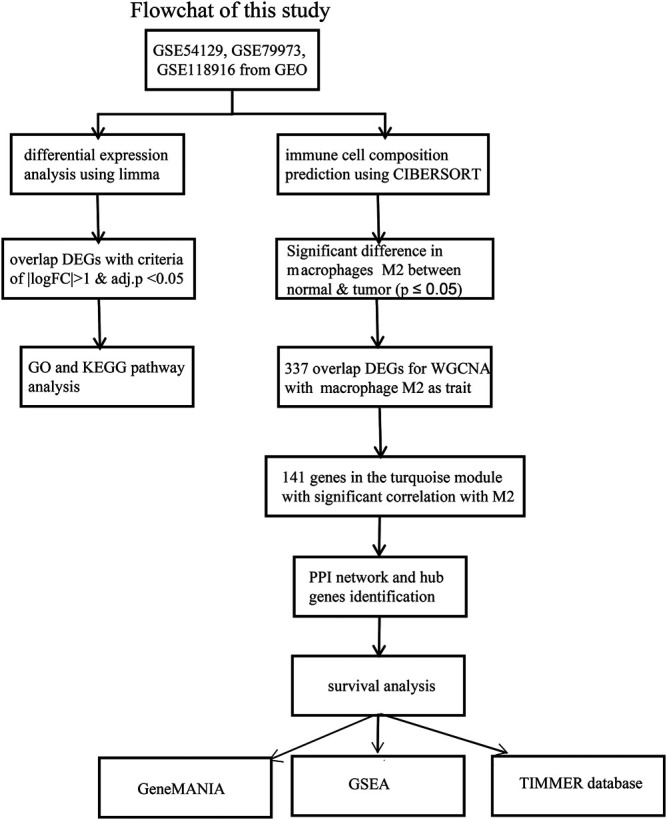
The workflow of the identification of hub genes related to Macrophage immune infiltration in GC.

Figure 1 showed the workflow of this study.

### DEG Identification and Functional Enrichment Analysis

**FIGURE 2:**
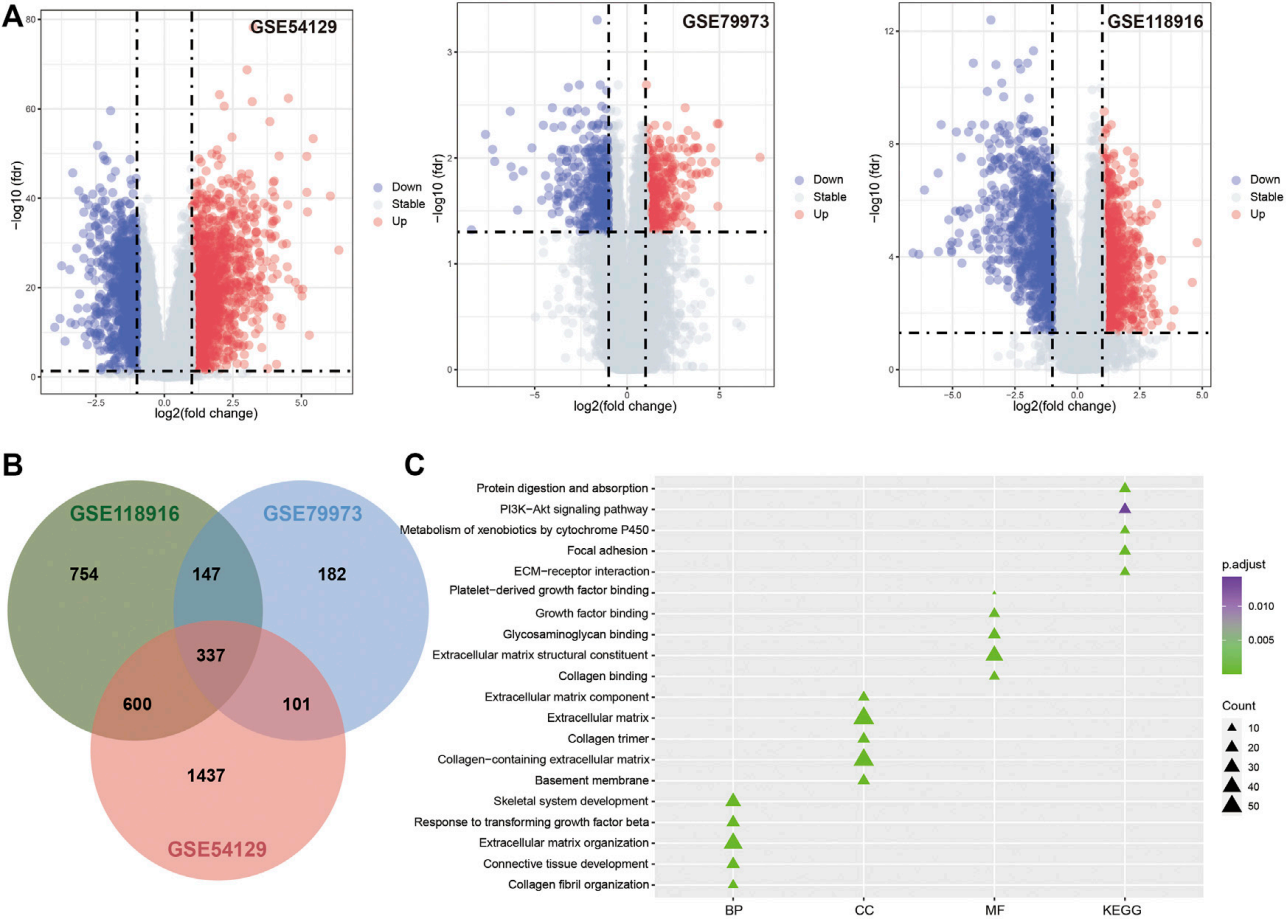
Differentially expression analysis results. **(A)**. Volcano plot for GSE54129, GSE79973 and GSE118916. **(B)**. The Venn diagram for DEGs identified in three GEO datasets. **(C)**. The functional enrichment analysis results for DEGs.

The microarray datasets for GC with the accession numbers GSE54129, GSE79973, and GSE118916 were used to identify DEGs respectively (Figure 2A; Table 1). A total of 337 overlapping genes were found from the three datasets (Figure 2B). GO function enrichment analysis revealed that the overlapping DEGs were engaged in biological processes, such as extracellular matrix organization, collagen catabolic process, and tissue development (Figure 2C); molecular functions, such as extracellular region, extracellular matrix, and collagen trimer (Figure 2C); and cellular components, such as extracellular matrix structural constituent and growth factor binding (Figure 2C). KEGG pathway analysis revealed that the DEGs were engaged in pathways of extracellular matrix (ECM)–receptor interaction, protein digestion and absorption, focal adhesion, xenobiotic metabolism by cytochrome P450, and chemical carcinogenesis (Figure 2C).

### Macrophage-Associated Module and Hub Genes Identification by WGCNA

**FIGURE 3:**
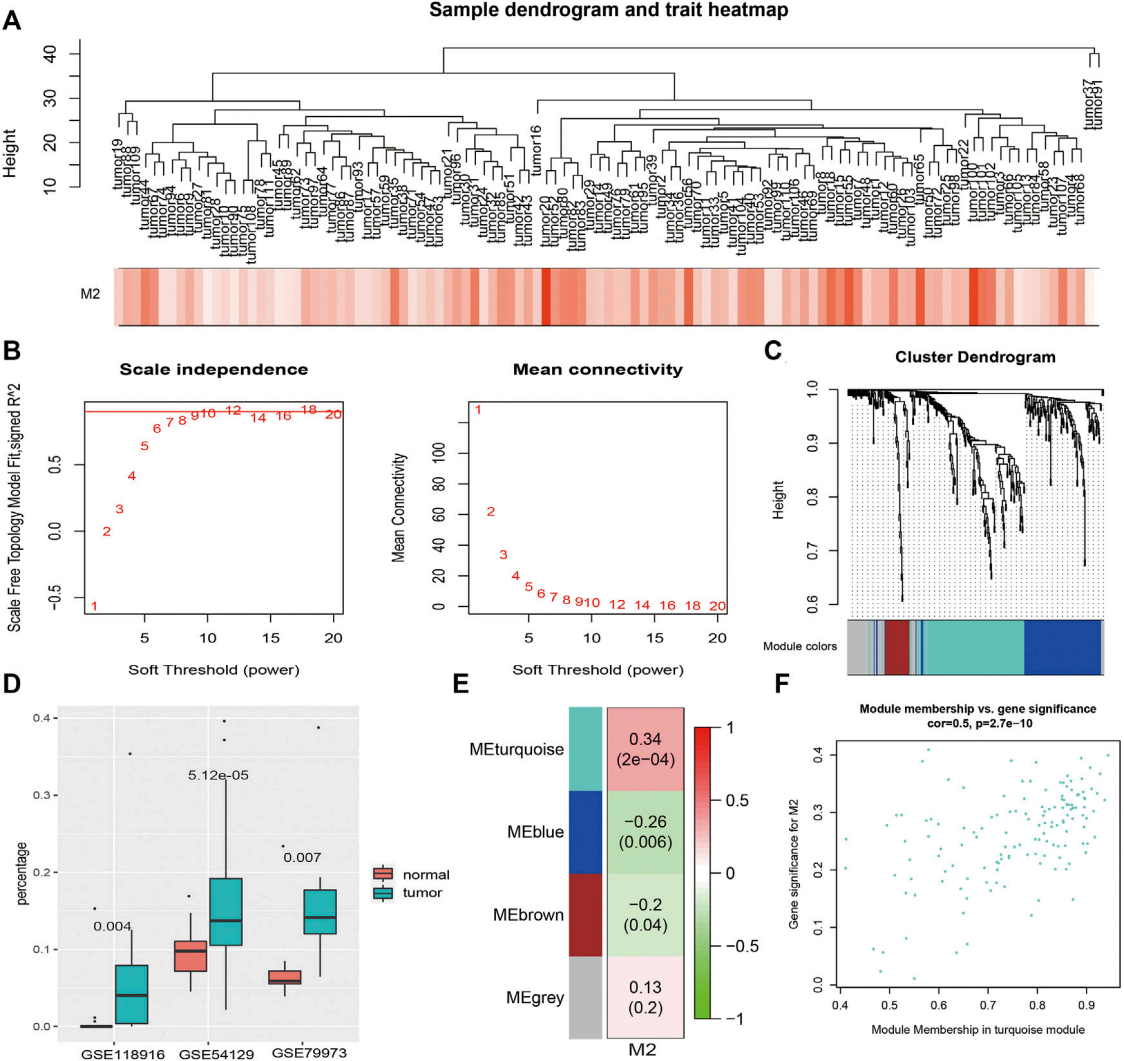
WGCNA results. **(A)**. Sample clustering with macrophage M2 as the external trait. **(B)**. WGCNA power selection. **(C)**. Dendrogram of the WGCNA modules. **(D)**. The boxplot of macrophage M2 percentages between GC patients and controls in three datasets. **(E)**. The relationship between coexpression modules and external traits. **(F)**. The scatter plot of MM and GS in the turquoise module.

M2 macrophages, which present statistical significance between GC patients and controls, along with 337 overlapping DEGs expression profiles in 111 GC patients in GSE54129 (Figure 3A) were included in the construction of coexpression network with 12 as the soft thresholding power β (Figure3B). Three modules were identified (Figure 3C). The immune infiltrating abundance of 22 immune cells were predicted using the three GEO datasets. As shown in Figure 3D, there were significant differences for M2 macrophages between GC patients and controls across the three datasets using Mann–Whitney U test with p value <0.01. Then the relationships between M2 macrophages and the three coexpression modules were explored in Figure 3E, and the result showed that M2 macrophages was most positively associated with the turquoise module. The association between MM and GS in the turquoise module was then analyzed (Figure 3F), which showed that GS in the M2 macrophages was significantly related to corresponding MM.

### Hub Genes Identification

The PPI network was built using the STRING database with 690 edges and 110 nodes. CytoHubba was used to filter hub genes in the PPI network. The top 25 hub genes were identified (Supplementary Table S1) in which seven of them were also screened out in the turquoise module. These fundamental genes include *COL1A1*, *COL4A1*, *COL5A2*, *COL12A1*, *LUM*, *PDGFRB*, and *THBS1*.

### Survival Analysis of the Hub Genes Using GEPIA and KM-Plotter

**FIGURE 4:**
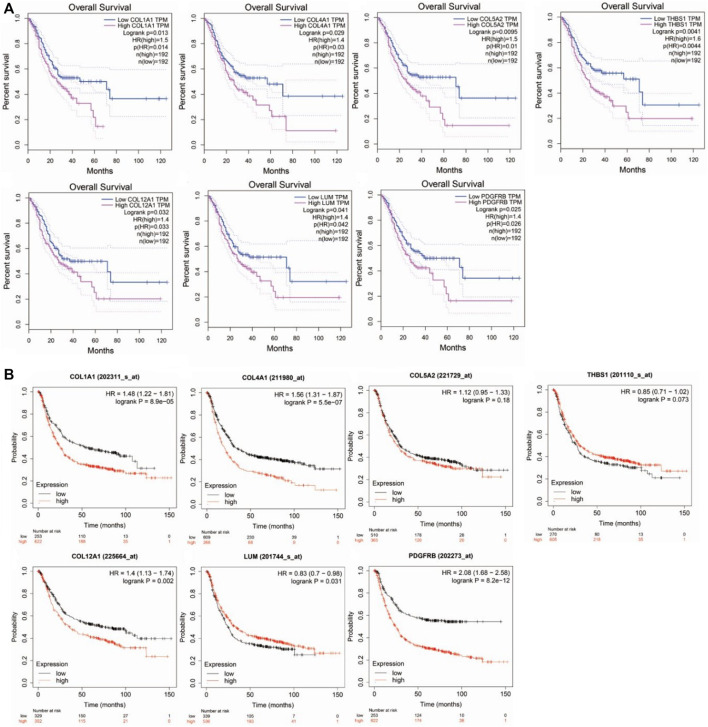
Survival analysis of hub genes in Gastric cancer. **(A)**. by GEPIA using TCGA datasets. **(B)**. by KM-plotter using microarray datasets.

The overall survival rate and median survival time of the patients with GC in the group with low hub gene expression were significantly higher than those in the group with the high hub gene expression, as demonstrated by GEPIA (Figure 4A). GEPIA predicts the survival rates for genes by using the RNA-seq data in TCGA. We further performed survival analysis by the KM plotter by using microarray datasets to validate the results of the GEPIA. As shown in Figure 4B, *COL1A1* (logrank *p* = 8.9e−5), *COL4A1* (logrank *p* = 5.5e−07), *COL12A1* (logrank *p* = 0.002), and *PDGFRB* (logrank *p* = 8.2e−12) were consistent with the results from the GEPIA and were identified as the hub genes for GC.

### Association of the Expression of Hub Genes With Tumor Purity and Immune Infiltration

**FIGURE 5:**
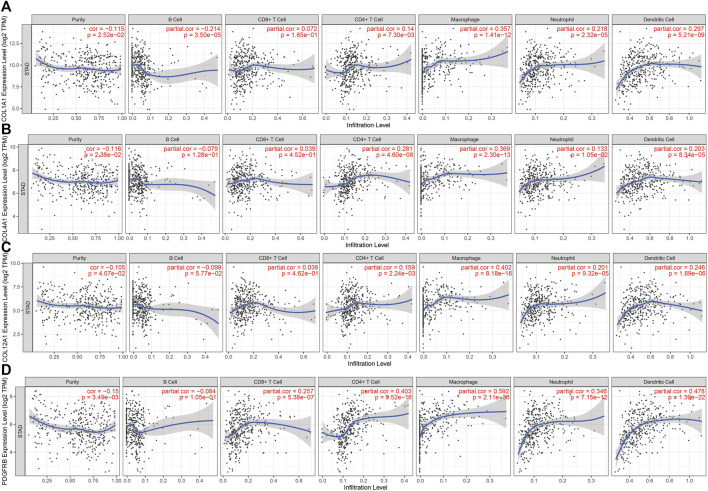
Correlation between hub genes expression and immune cell infiltration in STAD in the TCGA cohort. **(A)**. COL1A1 **(B)**. COL4A1 **(C)**. COL12A1 and **(D).** PDGFRB.

There are tumor cells, stromal cells, and infiltrating immune cells in the tumor microenvironment. TIMER was conducted to investigate the associations between the genes’ expression level in GC and both tumor purity and immune cell infiltration. The results revealed that *COL1A1*, *COL4A1*, *COL12A1*, and *PDGFRB* were all negatively correlated with tumor purity. Significant correlations were observed between these four genes and the infiltration of CD4^+^ T cells, macrophages, neutrophils, and dendritic cells (Figure 5A–D).

### Functional Analysis for Hub Genes

**FIGURE 6:**
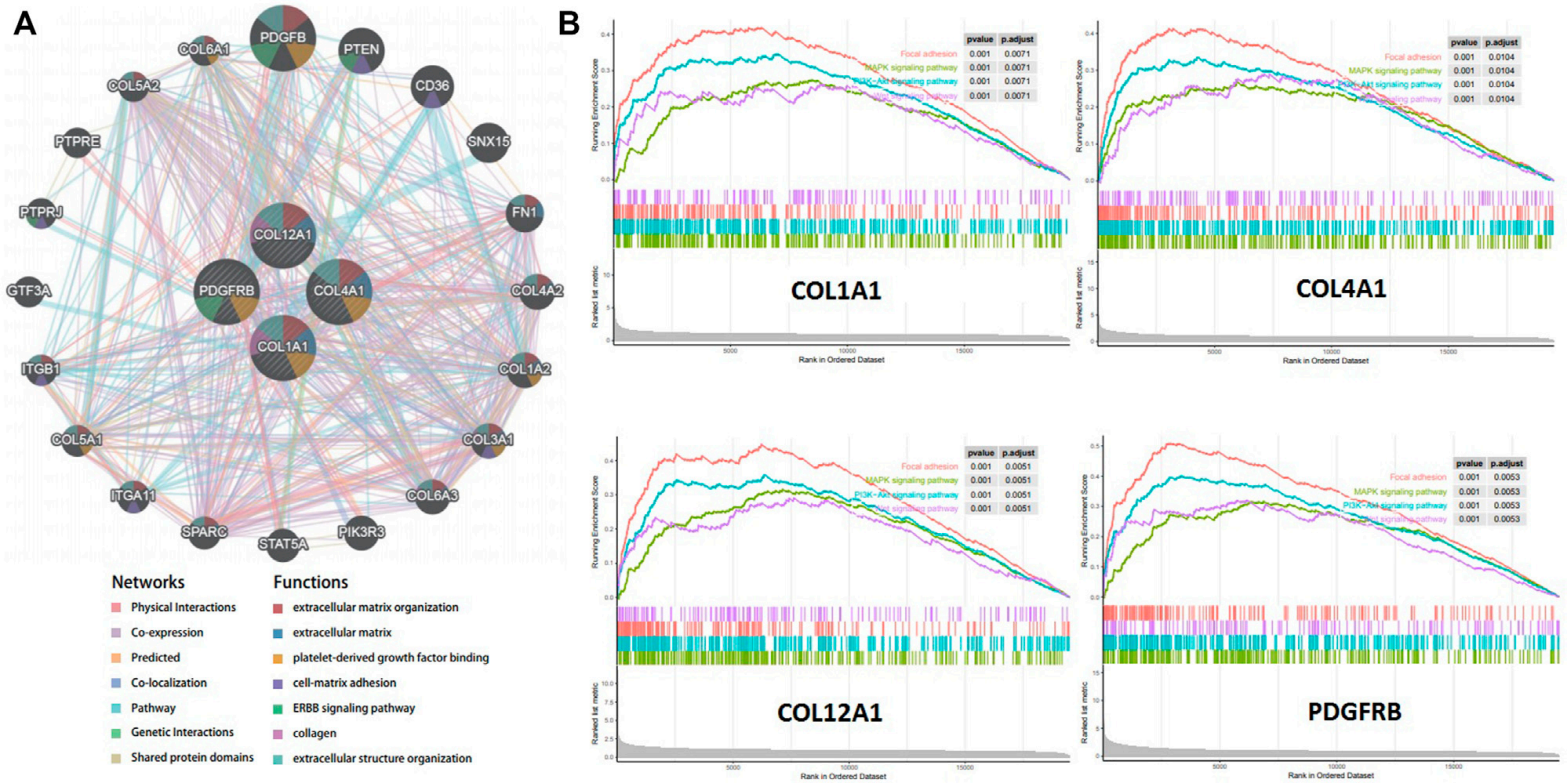
Function Prediction for hub genes. **(A)**. Protein-protein interaction network (geneMANIA) of gastric cancer related hub genes. **(B)**. Gene set enrichment analysis (GSEA) of hub genes in the TCGA-STAD dataset.

A gene interaction network was constructed to decipher the biological functions of these hub genes using GeneMANIA. Twenty genes associated to the four hub genes were identified, and further results showed that they were involved in extracellular matrix, cell–matrix adhesion, and ERBB signaling pathway (Figure 6A). To further explore the functions of the crucial genes in GC, we performed GSEA on the TCGA-STAD RNA-seq data. As shown in Figure 6B, genes in the high expression groups, namely, *COL1A1*, *COL4A1*, *COL12A1*, and *PDGFRB*, were all enriched in the MAPK and PI3K–Akt signaling pathways, which are closely associated with tumor cell proliferation, invasion, and cell cycle.

## Discussion

GC remains to be one of the most common cancers with high morbidity and mortality. Investigating gene biomarkers related to GC progress will be beneficial to improve the diagnostic accuracy and reduce the economic burden of patients. In the present study, a series of bioinformatics methods was performed to identify the biomarkers GC diagnosis and/or therapy. GO term and KEGG pathway analyses showed that the DEGs are involved in extracellular matrix organization, tissue development, blood vessel development, ECM–receptor interaction, focal adhesion, and protein digestion and absorption (Fischer et al., 2001; Abed Kahnamouei et al., 2020). Four hub genes of *COL1A1*, *COL4A1*, *COL12A1*, and *PDGFRB* were revealed to be significantly associated with patient outcomes.

Genes of *COL1A1, COL4A1*, and *COL12A1* are all related to ECM and collagen. Collagen, the major component of ECM, which plays an active role in many biological processes, including cell shape, proliferation, migration, differentiation, apoptosis, and carcinogenesis (Fischer et al., 2001). *COL1A1*, a type I collagen, is a main component for the family of fibrillar collagen and is engaged in the tumor invasion and progression (Li et al., 2016). Li et al. (2019b) demonstrated that *COL1A1* is overexpressed in GC and can be used to monitor early GC progression; furthermore, a high expression of *COL1A1* may serve as a prognostic factor predicting patients’ overall survival time. *COL4A1* is a collagen type IV and has the potential for promoting gastric carcinoma recurrence (Désert et al., 2016). Upregulation of *COL4A1* is related to advanced tumor stage and bad overall and disease-free survival in HCC patients (Salem et al., 2016). Storlazzi et al. (2006) validated that *COL4A1* knockdown can lead to the reduction of cell viability and cell cycle arrest in breast cancer cells. *COL12A1* has been suggested to be associated with various cancers, including subungual exostosis, ovarian, breast, and colon cancer, indicating that *COL12A1* may serve as a new potential biomarker for cancers (Sun et al., 2015). Recently, *COL12A1* has been reported as a potential biomarker for GC (Jiang et al., 2019). Januchowski et al. (2016) demonstrated that *COL12A1* is involved in the drug resistance of cancer cells and tumor progression. Our survival analysis results showed that a high mRNA level of *COL12A1* is in association with the poor prognosis in GC, and *COL12A1* may act as a potential biomarker in GC (Duan et al., 2018).

*PDGFRB* encodes for platelet-derived growth factor receptor beta, a typical transmembrane receptor tyrosine kinase (Steller et al., 2013). Numerous important biological processes, including growth, proliferation, movement, and survival, are controlled by *PDGFRB* (Kim et al., 2012), and its dysregulation is related closely to carcinogenesis (Heldin, 2013). Wallmann et al. (2018), showed that *PDGFRB* expression can stimulate the migratory capacity of glioma cells. In addition, a high expression level of *PDGFRB* in tumor stroma is closely related to large tumor size, advanced stage, high Gleason score, and high vessel density. Furthermore, high *PDGFRB* expression in the stroma of tumor and non-malignant tissue is in association with the short cancer-specific survival in prostate cancer patients (Hägglöf et al., 2010).

We also referred to TIMER and geneMANIA, and performed GSEA to explore the functions of the four hub genes in GC. The expression of *COL1A1*, *COL4A1*, *COL12A1*, and *PDGFRB* were all negatively correlated with tumor purity. Significant correlations were observed between these four hub genes and the infiltration of CD4^+^ T cells, macrophages, neutrophils, and dendritic cells, suggesting that the hub genes were likely related to tumor cell invasion into the surrounding microenvironment. The results of geneMANIA and GSEA showed that the upregulation of these hub genes is in association with the MAPK and PI3K/AKT signaling pathways and Wnt signaling pathways which contribute to GC proliferation and invasion (Singh et al., 2015; Lin et al., 2020).

## Conclusion

In summary, this study identified common DEGs by integrating three different GEO datasets between normal gastric tissues and GC tissues. Then, a series of bioinformatics methods was applied to these DEGs, including the associated signaling pathways and crucial genes from the PPI network and WGCNA modules, which may play vital roles in the carcinogenesis and development of GC. Furthermore, the hub genes can also immunologically regulate the tumor microenvironment. GSEA suggested their potential contribution to the pathogenesis of GC. These findings will shed light on the clarification of biological mechanisms and provide new biomarkers for GC.

## Data Availability

The datasets presented in this study can be found in online repositories. The names of the repository/repositories and accession number(s) can be found in the article/Supplementary Material.

## Footnotes

1
https://www.ncbi.nlm.nih.gov/geo/

2
http://www.genemania.org

3
http://cibersort.stanford.edu/

4
http://string-db.org

5
https://cistrome.shinyapps.io/timer/

6
http://gepia.cancer-pku.cn

7
http://kmplotter.com/analysis
