# Supplementary material for: Identification of Potential Prognostic Biomarkers Associated With Macrophage M2 Infiltration in Gastric Cancer
Source: Front Genet. 2022 Jan 17;12:827444. doi: 10.3389/fgene.2021.827444 (PMC8802722; doi:10.3389/fgene.2021.827444)
Supplement: Supplementary file 1 [file Table1.DOC]

| name | Betweenness | BottleNeck | Closeness | ClusteringCoefficient | Degree | DMNC | EcCentricity | EPC | MCC | MNC | Radiality | Stress |
| --- | --- | --- | --- | --- | --- | --- | --- | --- | --- | --- | --- | --- |
| COL3A1 | 3548.55599 | 8 | 125.78333 | 0.34906 | 53 | 0.56348 | 0.15679 | 156.781 | 3.17E+12 | 53 | 8.8794 | 31080 |
| COL1A1 | 4537.89274 | 5 | 130.43333 | 0.30625 | 59 | 0.51154 | 0.15679 | 156.781 | 3.17E+12 | 59 | 8.95377 | 42540 |
| COL1A2 | 2616.81229 | 48 | 124.85 | 0.38531 | 50 | 0.61051 | 0.15679 | 156.781 | 3.17E+12 | 50 | 8.89056 | 30840 |
| COL4A1 | 947.2042 | 3 | 111.71905 | 0.57765 | 33 | 0.84245 | 0.13439 | 156.781 | 3.17E+12 | 32 | 8.65258 | 10074 |
| COL6A3 | 767.92168 | 3 | 111.3 | 0.61089 | 32 | 0.88334 | 0.15679 | 156.781 | 3.17E+12 | 31 | 8.67489 | 7890 |
| COL4A2 | 597.84117 | 1 | 112.05238 | 0.56439 | 33 | 0.78116 | 0.13439 | 156.781 | 3.17E+12 | 33 | 8.66374 | 8496 |
| COL5A1 | 444.98731 | 7 | 113.26905 | 0.54444 | 36 | 0.7755 | 0.13439 | 156.781 | 3.16E+12 | 36 | 8.67118 | 6660 |
| COL6A2 | 619.35101 | 3 | 110.08571 | 0.55847 | 32 | 0.80754 | 0.13439 | 156.781 | 2.99E+12 | 31 | 8.6154 | 6514 |
| COL5A2 | 576.68542 | 4 | 114.18333 | 0.51351 | 37 | 0.73805 | 0.15679 | 156.781 | 2.99E+12 | 37 | 8.70836 | 10024 |
| FN1 | 16573.96244 | 143 | 141.23571 | 0.20523 | 71 | 0.37231 | 0.13439 | 156.781 | 2.99E+12 | 70 | 9.11366 | 87968 |
| BGN | 4155.87526 | 7 | 121.66667 | 0.36039 | 46 | 0.62347 | 0.15679 | 156.781 | 2.99E+12 | 43 | 8.8385 | 30552 |
| COL11A1 | 159.92831 | 1 | 108.16905 | 0.66667 | 28 | 0.87344 | 0.13439 | 156.781 | 2.99E+12 | 28 | 8.60425 | 3498 |
| LUM | 3035.04157 | 10 | 112.75 | 0.55444 | 32 | 0.80171 | 0.15679 | 156.781 | 2.98E+12 | 31 | 8.73439 | 23664 |
| FBN1 | 2203.41787 | 8 | 113.36905 | 0.50588 | 35 | 0.74998 | 0.13439 | 156.781 | 2.97E+12 | 34 | 8.68977 | 14894 |
| THBS2 | 917.25714 | 1 | 112.33571 | 0.47594 | 34 | 0.66527 | 0.13439 | 156.781 | 2.81E+12 | 34 | 8.67489 | 10064 |
| COL12A1 | 347.07871 | 1 | 111.41667 | 0.57008 | 33 | 0.78903 | 0.15679 | 156.781 | 1.67E+12 | 33 | 8.66374 | 5992 |
| SPARC | 2878.74233 | 7 | 117.05476 | 0.45377 | 38 | 0.68841 | 0.13439 | 156.781 | 1.50E+12 | 37 | 8.7567 | 22124 |
| COL8A1 | 15.59859 | 1 | 95.17857 | 0.90196 | 18 | 1.01371 | 0.13439 | 156.606 | 1.81E+11 | 18 | 8.28075 | 366 |
| THBS1 | 1073.33239 | 1 | 112.05238 | 0.49032 | 31 | 0.66469 | 0.13439 | 156.781 | 1.77E+11 | 31 | 8.70092 | 13920 |
| NID2 | 24.42737 | 1 | 101.82143 | 0.88235 | 17 | 0.97144 | 0.13439 | 156.654 | 1.75E+11 | 17 | 8.52616 | 344 |
| COL8A2 | 1547.41488 | 2 | 93.32143 | 0.83088 | 17 | 1.01409 | 0.13439 | 156.781 | 1.74E+11 | 16 | 8.22126 | 27022 |
| COL10A1 | 369.85019 | 1 | 97.0381 | 0.63684 | 20 | 0.81078 | 0.13439 | 156.781 | 1.74E+11 | 19 | 8.32165 | 3508 |
| SERPINH1 | 184.92871 | 3 | 104.03571 | 0.72857 | 21 | 0.86481 | 0.13439 | 156.781 | 9.97E+10 | 21 | 8.55219 | 3192 |
| P4HA3 | 31.96362 | 1 | 98.08571 | 0.97143 | 15 | 1.02152 | 0.13439 | 156.286 | 8.77E+10 | 15 | 8.42948 | 1076 |
| PDGFRB | 1035.80716 | 1 | 110.55476 | 0.4418 | 28 | 0.57883 | 0.13439 | 156.781 | 6.75E+09 | 28 | 8.66374 | 9688 |
